# The Therapeutical Action of the Salts of Strontium

**Published:** 1892-07-02

**Authors:** 


### THERAPEUTICS.

### THE THERAPEUTICAL ACTION OF THE SALTS OF STRONTIUM.

The strontium salts were for a long time disregarded for therapeutical purposes, and until recently were thought to be poisonous. Thanks to the investigations of M. Laborde, such an impression has been shown to be erroneous, and more recently still the usefulness of strontium salts have been amply established by many observers. As these results, however, are but little known to English practitioners, it will be well to make full reference to some of the more important communications on strontium. M. Laborde's experiments on dogs demonstrated that the strontium salts were entirely innocuous, and that they were effective diuretics, and stimulated the gastric secretion.

Dr. Bardet,* like most observers, lays great stress on the necessity of obtaining the perfectly pure salt, and this point requires attention, inasmuch as the strontium salts are so liable to be contaminated with barium. Strontium and barium are always found associated in nature, and it is difficult to obtain a salt of strontium which does not give the spectroscope of barium. Yet, although barium may be toxic, we must not forget that this metal has been employed in doses of half to one gramme by men of such high standing as Lisfranc and Baudelocque, who have obtained very good results thereby in the treatment of scrofula. This remedy has been replaced by iodine, but it should, nevertheless, be retained in our pharmacopœia. It would appear, then, that the necessity for chemical purity of the strontium salt has been exaggerated.

To test the salts of strontium we take a gramme of the salt and dissolve it in ten grammes of distilled water. To this solution add one cubic centimeter of a ten per cent. solution of bichromate of potassium. The mixture ought to remain clear. If a cloudiness appears it is due to the presence of appreciable traces of a barium salt in the salt under examination.

As regards the pharmacology of strontium, Dr. Bardet states that up to the present time the bromide, iodide, chloride, lactate, and the nitrate have been employed. To obtain the bromide we act on strontium or its pure carbonate with hydrobromic acid, when by evaporation of the liquid hygroscopic crystals are thrown down, very soluble in water and less so in alcohol, having a saltish and characteristic taste of bromides. This salt is given in the usual manner with bromides, either in solution or in pill. The iodide may be obtained from sulphide of strontium by mixing a solution of it with tincture of iodine. The lactate is formed by saturating lactic acid with pure carbonate of strontium. An amorphus granular salt is formed. He gives the following table of solubility of the salts of strontium. One part by weight dissolves at 20 deg. Cent. thus :—

| | | |
|---|---|---|
| Bromide | in 1·01 | times its weight of water. |
| Chloride | 1·89 | ,, ,, ,, |
| Iodide | 0·56 | ,, ,, ,, |
| Nitrate | 5·00 | ,, ,, ,, |
| Strontian | 52·00 | ,, ,, ,, |

The carbonate, sulphate, and the phosphate of strontium are nearly or quite insoluble in water. In prescribing the carbonate we obtain the same result as with the chloride or lactate, since the carbonate becomes transformed in the stomach into one or the other of the latter by the action of the free acid of the gastric juice.

*Physiology of Strontium.*—We have already said that the salts of strontium are non-toxic, but this was far from being generally recognised, and it was the experiments of Laborde which showed that, not only are they innocuous, but that their entire absence from toxic quality is more marked than is the case with the salts of potassium, sodium, and ammonium. Laborde showed that we could cause a considerable quantity of strontium salts to enter the circulation, even by intravenous injection, without harm ensuing. And as regards human beings, he was able to prove that a healthy individual could absorb with impunity the chloride and nitrate, and that, as a matter of fact, in a number of instances the only result appeared to be great improvement in appetite.

Therapeutically, the bromide has been employed by Féré

* "Les Nouveaux Remèdes," No. 3, 1892.

in the large dose of fifteen grammes, without the occurrence of intolerance such as he had observed with bromide of potassium. Germain Sée has found that small doses of bromide of strontium increase the appetite, and have a good effect in certain classes of atonic dyspepsia. He used as a minimum dose thirty grains, and as a maximum dose sixty grains, but his observations require confirmation. The salt was found to be more potent than sodium, and was administered in thirty-two cases of indigestion in which there was hyperacidity, or dilatation of the stomach with pain and marked flatulency. The digestion for proteids was complete, but not so for fats and starch. In every instance the symptoms were rapidly alleviated, and in many cases a complete cure resulted. Bromide of strontium is equally useful in gastralgia, and it yields good results in heart disease and in Bright's disease.

M. Constantin Paul, as far back as July, 1891, communicated some results he had obtained with strontium as a diuretic, and more recently he has confirmed his earlier observations by subsequent investigations. He employed the lactate in his earlier cases, though more recently he has given the bromide and the nitrate. In speaking of its use in Bright's disease this author states that the lactate of strontium is not diuretic, but it notably diminishes the amount of albumen in the urine, showing a marked amelioration in the other symptoms. When the strontium was discontinued the albumen reappeared on the following day, to disappear once more on resuming the drug. Although the number of cases under treatment are too small for M. Paul to speak definitely as to its value, he considers the results hitherto are most encouraging. One of his cases reported* was that of a man, aged 41, with chronic parenchymatous nephritis, and passing a very large amount of albumen. This man was passing about a litre of urine daily; he had a moderate degree of anasarca. There were rales audible all over both sides of the chest. He suffered from palpitation, and there was a *bruit de galop* characteristic of ventricular hypertrophy. He had loss of appetite, headache, and suffered from cramp. The urine being somewhat deficient, he was ordered thirty centigrammes of powdered digitalis. This treatment was continued four days, but as polyuria was not induced, the administration of digitalis was stopped, and the patient was henceforth put exclusively on a milk diet; he took almost three litres daily. However, the amount of albumen had fallen in the four days from 7½ to 3½ grammes. For six days following the administration of digitalis, the urine did not increase, but remained stationary at one litre, the albumen likewise remained at 3½ grammes daily. On June 4th the administration of lactate of strontium was begun in ten gramme daily doses. For the three first days the urine increased and reached almost to three litres, but the albumen fell from 3½ grammes to 1·80 grammes, and two days later it had fallen to 20 centigrammes daily; the urine fell and remained stationary at 2½ litres daily.

On the twelfth day, after the treatment had been continued for eight days, the patient was very greatly improved, and this improvement continuing, one was led to believe in the possibility of leaving off the drug. He had taken 10 grammes daily for eleven days, but the day following the omission of the strontium the albumen increased from 10 centigrammes to 1·20 grammes, so that two days later the drug was once more resumed and steadily persevered in till July 4th. At that period the urine only contained traces of albumen, about 0·50 gr. daily. Then on leaving off the drug the albumen did not again increase, and the patient feeling well asked to be allowed to leave the hospital on July 18th.

This case affords several points of interest, viz.: (1) at first the diminution in the amount of albumen was the result of being under hospital regimen, rather than brought about by the digitalis maceration, which apparently accomplished nothing at all; (2) the complete tolerance of lactate of strontium administered for thirty-eight days in daily amounts of ten grammes; (3) the rapid fall in the amount of albumen almost from the commencement of the treatment by strontium, but a diminution which never touched zero point; (4) the necessity of continuing the administration of the strontium for ten days after the fall in albumen, without which one observes the quantity increase again immediately from the day following the discontinuation of the remedy; (5) finally, the amelioration of all the symptoms accompanying the fall in albumen, so much so that the patient could leave the hospital in excellent condition, although he still passed about half a gramme of albumen. We cannot possibly give the details of M. Paul's other interesting cases, but we may give his conclusions based on his extended observations. He believes that strontium is of value for the relief of albuminuria under the following conditions, viz., in rheumatic parenchymatous nephritis, and in low parenchymatous nephritis of scrofula and gout. He has also found it useful in the albuminuria of pregnant women, and in those recently delivered. It is, however, of slight value only in the albuminuria of syphilis, in infectious albuminuria, and in that due to cardiac disease. In interstitial nephritis, and in the albuminuria of tuberculous cachexia it is useless. The diminution of the albumen in all cases is followed by a very noticeable amelioration of the other symptoms. If the salts of strontium are to be useful in albuminuria it is necessary that they be employed before the period in which urinary insufficiency and uræmia develop. A febrile state or high temperature contra-indicates the administration of lactate of strontium in parenchymatous nephritis.

* "Les Nouveaux Remèdes," Dec. 8th, 1891.

